# Social Support and Symptom Severity Among Patients With Obsessive-Compulsive Disorder or Panic Disorder With Agoraphobia: A Systematic Review

**DOI:** 10.5964/ejop.v14i1.1252

**Published:** 2018-03-12

**Authors:** Véronique Palardy, Ghassan El-Baalbaki, Catherine Fredette, Elias Rizkallah, Stéphane Guay

**Affiliations:** a Department of Psychology, Université du Québec à Montréal, Montreal, Canada; b Faculty of Medicine, McGill University, Montreal, Canada; c Department of Sociology, Université du Québec à Montréal, Montreal, Canada; d School of Criminology, Université de Montréal, Montreal, Canada; e Institut Universitaire en Santé Mentale de Montréal, Montreal, Canada; Department of Psychology, Webster University Geneva, Geneva, Switzerland; Psychology Department, College of New Rochelle, New Rochelle, NY, USA

**Keywords:** obsessive-compulsive disorder, panic disorder, agoraphobia, social support, marital adjustment, accommodation, expressed emotion

## Abstract

Panic disorder with or without agoraphobia (PD/A) and obsessive-compulsive disorder (OCD) are characterized by major behavioral dysruptions that may affect patients’ social and marital functioning. The disorders’ impact on interpersonal relationships may also affect the quality of support patients receive from their social network. The main goal of this systematic review is to determine the association between social or marital support and symptom severity among adults with PD/A or OCD. A systematic search of databases was executed and provided 35 eligible articles. Results from OCD studies indicated a negative association between marital adjustment and symptom severity, and a positive association between accommodation from relatives and symptom severity. However, results were inconclusive for negative forms of social support (e.g. criticism, hostility). Results from PD/A studies indicated a negative association between perceived social support and symptom severity. Also, results from studies using an observational measure of marital adjustment indicated a negative association between quality of support from the spouse and PD/A severity. However, results were inconclusive for perceived marital adjustment and symptom severity. In conclusion, this systematic review generally suggests a major role of social and marital support in PD/A and OCD symptomatology. However, given diversity of results and methods used in studies, more are needed to clarify the links between support and symptom severity among patients with PD/A and OCD.

Anxiety disorders are the most common psychiatric disorders (Antony, 2011), with a 4.5% prevalence in the world population (Vos et al., 2012). Panic disorder (PD) is characterised by recurrent panic attacks and persistent concern about the attacks or their consequences (APA, 2013). A disorder commonly diagnosed with panic disorder is agoraphobia (A), which is anxiety about being in places or situations in which escape might be difficult or help might not be available in case of an attack, and often lead to complete or partial avoidance of the anticipated situations (APA, 2013). Obsessive-compulsive disorder (OCD) is characterised by recurrent obsessions (persistent and intrusive thoughts, ideas, impulses or images) that cause anxiety, and compulsions (repetitive behaviors or mental acts) that are performed in order to prevent or decrease obsessions-related anxiety (APA, 2013). Although OCD is not categorized as an anxiety disorder in the latest version of the DSM (DSM-5; APA, 2013), anxiety is still considered one of its major components. Moreover, the present review covers the time period when OCD definition was based on the criteria from the third and fourth versions of the DSM (APA, 1980; APA, 1994), which still classified OCD as an anxiety disorder.

OCD and PD/A share common aspects in addition to anxiety. Indeed, these disorders both include major behavioral disruptions that can heavily affect patients’ social and marital functioning (Markowitz, Weissman, Ouellette, Lish, & Klerman, 1989). Researchers investigating the impact of mental disorders discovered that it was distressing not only for the patients but for the family as well (Maurin & Boyd, 1990), which is partly caused by caregiving responsibilities toward the affected person (Maurin & Boyd, 1990). It can thus be expected that the relatives’ burden will affect the support that they provide to the suffering person. The following will present different concepts that are of importance when assessing social support in relation to OCD and PD/A.

## Social Support

Social support is defined as the process through which help is provided or exchanged with others in an attempt to facilitate one or more adaptational goals (Cohen, Gottlieb, & Underwood, 2000). Social support is a complex and multifaceted construct that can be broken down into different types. Cohen (1992) distinguishes between perceived social support, received social support, and social networks. Perceived and received social support both concern the quality of social support, whereas the assessment of social networks provides a more quantitative description of social support. More specifically, perceived social support refers to the respondent’s perception that his or her relationships will provide resources such as emotional support and information, and can be assessed with self-report questionnaires or interviews. Received social support refers to supportive behaviors that a person does to help another face stressful life events. This type of support is observed or assessed in a more objective way, for example by asking if a specific supportive action has been performed (Helgeson, 1993). Also, this measure does not take into account the perception of the person receiving or providing the supportive behaviors. Although social networks provide information about the existence, quantity, and types of social relationships, they appear to be less associated with wellbeing than are perceived and received social support (Cohen & Wills, 1985). Indeed, many studies have shown a negative association between quality of social support and psychological distress (Brown, Andrews, Harris, Adler, & Bridge, 1986; Cramer, 1991; Krause, Liang, & Yatomi, 1989; Panayiotou & Karekla, 2013).

In addition to the different types of support mentioned above, there are two dimensions in social support: positive and negative (Ray, 1992). Positive social support concerns positive attitudes and behaviors from one person toward another, for example self-disclosure and validation (Pizzamiglio, Julien, Parent, & Chartrand, 2001; Ray, 1992). Manifestations of negative social support include irritation, frustration, critical comments, conflicts, misunderstanding and negative pressure from others (Ray, 1992).

## Marital Adjustment

Support can be provided by different sources. Although friends and family can provide emotional or instrumental support, the spouse or partner is generally considered as the principal source of support (Boeding et al., 2013; Brown & Harris, 1978; Caplan, 1974; Cutrona & Russell, 1990; Jacobson, Holtzworth-Munroe, & Schmaling, 1989). When looking at support between spouses or partners, marital adjustment is of interest. It refers to the quality of the marital relationship and is comprised of four components: troublesome dyadic differences, interpersonal tensions and personal anxiety, dyadic satisfaction and cohesion, and consensus on matters of importance to marital functioning (Spanier, 1976). It is of note that marital adjustment is a concept wider than marital support. However, given that most marital adjustment questionnaires assess some aspects of marital support (for example “do you confide in your partner”), it cannot be disregarded when looking at marital support. Also, results from El-Baalbaki and colleagues (2011) have shown that a higher level of marital adjustment was associated with more displays of support and validation during a problem solving interaction between spouses. Since marital adjustment seems to be associated with an observational measure of marital support, it is likely that these two concepts share common aspects.

## Expressed Emotion

It is known that psychiatric disorders cause distress and dysfunction for people suffering from them. However, these conditions can also affect patients’ relatives and friends, who may develop negative attitudes toward the patient. Expressed Emotion refers to emotions a relative expresses about a psychiatric patient (Chambless, Bryan, Aiken, Steketee, & Hooley, 2001). This concept includes three dimensions: hostility, criticism and emotional over-involvement. Emotional over-involvement can be described as intrusiveness, excessively self-sacrificing behavior, or exaggerated emotional response to the patient’s illness (El-Baalbaki et al., 2011). Criticism and hostility refer to critical comments and negative attitudes toward the patient about the disorder and, as such, are manifestations of negative social support. Expressed Emotion is generally assessed during an interview with the relative alone, the Camberwell Family Interview (CFI; Vaughn & Leff, 1976). It can also be assessed with self-report measures, for example the Perceived Criticism Scale (PCS; Hooley & Teasdale, 1989) that assesses the relative’s level of criticism toward the patient, as perceived by the patient.

## Accommodation

Family accommodation is a term used to describe the behavioral involvement of patients’ relatives in some aspects of the disorder. An example would be the participation of a relative in the ritual of a patient suffering from OCD. This concept is often used in studies of OCD relatives, since they appear to be more involved in illness behaviors than relatives of patients with other mental disorders (Cooper, 1996). Studies have shown that accommodation is performed by more than 88% of OCD relatives (Calvocoressi et al., 1995; Calvocoressi et al., 1999; Stewart et al., 2008; Vikas, Avasthi, & Sharan, 2011) and that most of them accommodate on a daily basis (Stewart et al., 2008). Although participation in patients’ rituals is a frequent form of accommodation, relatives also accommodate by helping to avoid objects or places that exacerbate anxiety or by excessively reassuring the patient about the obsessions (Calvocoressi et al., 1995).

Accommodation behaviors are generally aimed to support or help the person with OCD (Boeding et al., 2013) and most relatives accommodate in order to decrease patients’ distress or anger (Calvocoressi et al., 1999). It is thus considered as a positive and specific form of social support, because the supportive behaviors specifically concern the symptoms of the disorder. However, accommodation might be associated with long-term negative outcomes. Although it may decrease immediate patients’ distress, it could maintain OCD symptoms by helping patients to avoid their anxiety, thus preventing them from becoming habituated to their fear and confronting their irrational beliefs (Salkovskis, 1996).

Accommodation is generally assessed with interviews or self-report questionnaires. The Family Accommodation Scale (FAS; Calvocoressi et al., 1999) is the most used instrument to assess the level of family accommodation and associated burden. It is a 12-item questionnaire administered by a clinician to relatives of OCD patients. A self-rated version of the FAS was also recently developed (Pinto, Van Noppen, & Calvocoressi, 2013).

## Objectives and Hypotheses

The main objective of this systematic review is to assess whether social and marital support is associated with severity of OCD and PD/A. It this hypothesized that positive social support will negatively correlate with severity of the disorders, whereas negative social support will correlate positively with severity of the disorders.

## Method

### Selection of Articles

The search covered Pubmed, PsycNET Proquest, CINAHL, Embase, ISI, SCOPUS, Cochrane databases, from January 1 1980 to June 30 2014, for articles concerning the association between social/marital support and severity of OCD or PD/A symptoms before any treatment. It has been decided to select articles published since 1980 given that anxiety disorders were officially recognized for the first time in the third version of the DSM (DSM-III; (APA, 1980), which was published in 1980. The generic query used was ("social support" OR accommodation OR "Expressed Emotion" OR spousal OR marital OR couple) AND ("panic disorder*" OR agoraphobia OR "obsessive-compulsive disorder*"). An independent search was also done in April 2016 to look for articles released since June 2014. All databases mentioned above were covered and the same generic query was used for the independent search.

### Inclusion/Exclusion Criteria

Eligible articles included any article in any language published in final form, even if the abstract was not available in English, and that assessed the association between social/marital support and severity of OCD or PD/A symptoms. Included studies were published after December 30 1979 and conducted among adult participants (18 years and over) with a primary diagnosis of OCD or PD/A. When possible, search limitations were set in order to only include articles dating from 1980 with participants aged 18 years and over. When this was not possible, manual selection was carried out. Any type of study was included, except case report studies. Finally, included studies measured severity of OCD or PD/A symptoms as well as social/marital support with self-reported questionnaires, observational instruments, or interviews by independent assessors.

### Selection Procedure

Two independent reviewers screened the studies for eligibility. If the title, abstract or keywords of the article contained clear indications that social or marital support was assessed, a full text review of the article was conducted. Any disagreement on eligibility of articles between reviewers after full text review was resolved by consensus after consultation with a third independent reviewer. Inter-rater reliability between the two independent correctors after full text review was 97% (171/177). For the remaining six articles, a consensus was reached between the reviewers (three were included and three were excluded).

## Results

**Figure 1:**
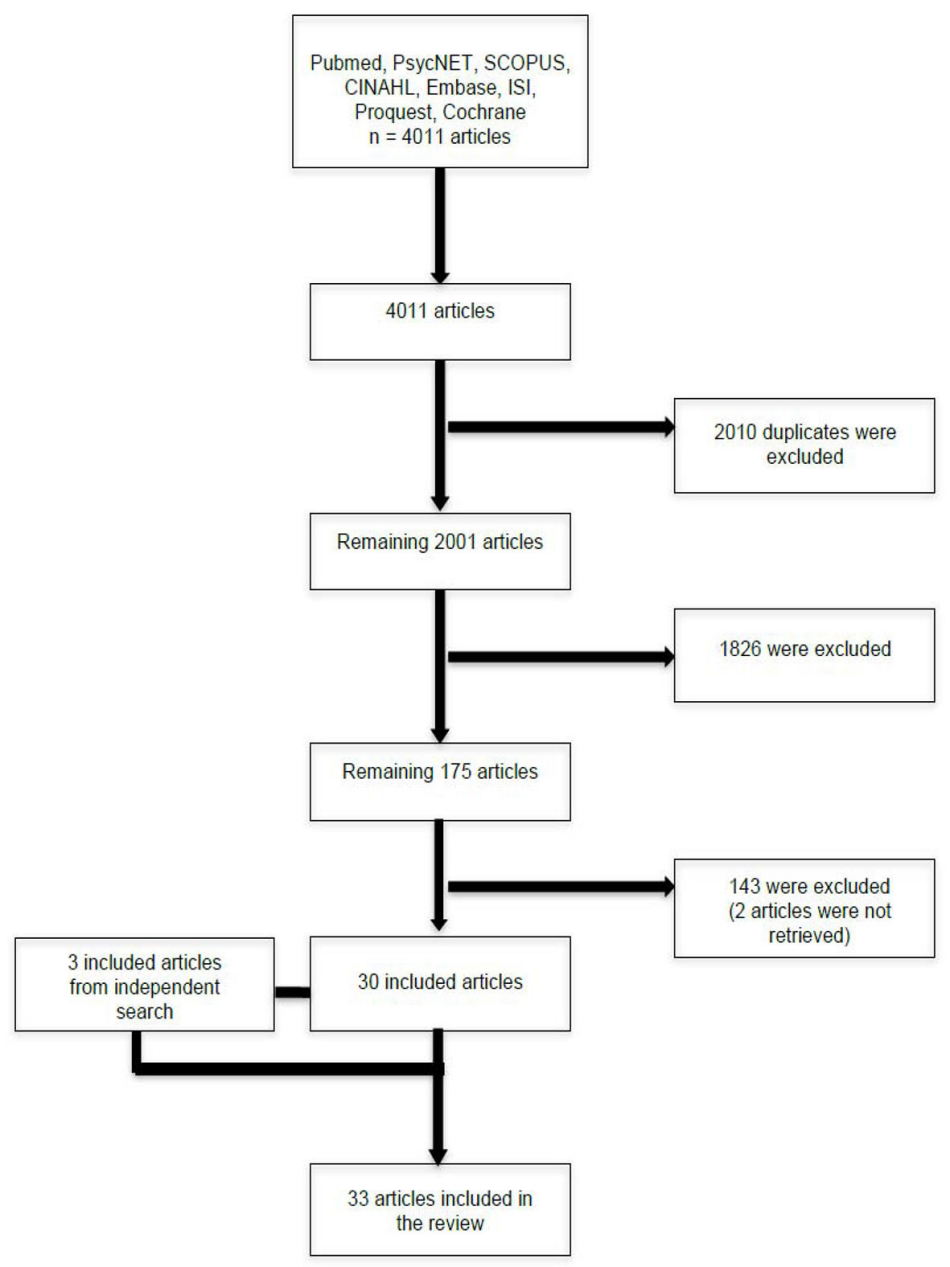
Article selection following each step.

The electronic database search provided 4011 articles, from which 2010 duplicates were removed. The other 2001 articles were screened by title, abstract and keywords, and this first selection led to the removal of 1826 articles. Two articles were impossible to retrieve (authors’ contacts could not be found; Cohen, 1986; Kitch, 1983), which led to 173 articles that went through a full text review. Among these articles, 148 were written in English, six in Chinese, six in German, four in French, three in Italian, two in Portuguese, one in Japanese, one in Korean, one in Turkish, and one in Dutch. Finally, 30 of these articles were included in the present review. Results from the independent search led to the inclusion of three other articles, for a total sample of 33 articles. Reasons for exclusion of the remaining 143 articles are described in Table 1. The article selection is described in detail in Figure 1.

**Table 1:** Reasons for Exclusion of 143 Articles After Full Text Review

Association not assessed between social or marital support and PD/A or OCD severity at pretreatment (Number of excluded articles: 71)
(Abramowitz et al., 2013; Albert, Brunatto, Aguglia, et al., 2009; Almasi, Akuchekian, & Maracy, 2013; Arnow, Taylor, Agras, & Telch, 1985; Arrindell & Emmelkamp, 1986; Barlow, Mavissakalian, & Hay, 1981; Barlow, O’Brien, & Last, 1984; Batinić, Trajković, Duisin, & Nikolic-Balkoski, 2009; Beekman et al., 1998; Byrne, Carr, & Clark, 2004; Cerny, Barlow, Craske, & Himadi, 1987; Chambless, Blake, & Simmons, 2010; Chambless, Bryan, Aiken, Steketee, & Hooley, 1999; Chambless & Steketee, 1999; Cheng, Jiang, & Du, 1998; Chernen & Friedman, 1993; Craske, Burton, & Barlow, 1989; Daiuto, 1996; De Berardis et al., 2008; El-Baalbaki, Bélanger, Perreault, Fredman, & Baucom, 2010; Emmelkamp, 1980; Emmelkamp, de Haan, & Hoogduin, 1990; Emmelkamp & de Lange, 1983; Emmelkamp et al., 1992; Emmelkamp & Gerlsma, 1994; Fauerbach, 1992; Ferrão et al., 2006; Fisher, 1983; Fisher & Terence Wilson, 1985; Friedman, 1990; Fukada, 2010; Grunes, 1998; Hafner, 1984; Himadi, Cerny, & Barlow, 1986; Hou, Yen, Huang, Wang, & Yeh, 2010; Huang, Li, Han, & Xiong, 2013; Jansson, Öst, & Jerremalm, 1987; Katerndahl, 2000; Katerndahl & Realini, 1997b; Keijsers, Hoogduin, & Schaap, 1994a, 1994b; Kleiner & Marshall, 1987; Koujalgi, Nayak, Patil, & Chate, 2014; Lebowitz, Panza, Su, & Bloch, 2012; Lelliott, Marks, Monteiro, Tsakiris, & Noshirvani, 1987; Löhr, Schewe, Baudach, & Hahlweg, 2003; Mannetter, 1989; Marchand et al., 1985; Marchand, Boisvert, Baudry, Berard, & Gaudette, 1984; McCarthy & Shean, 1996; Nauta, Batelaan, & Van Balkom, 2012; Oatley & Hodgson, 1987; Omranifard, Akuchakian, Almasi, & Maraci, 2011; Pace, Thwaites, & Freeston, 2011; Peter et al., 1998; Pyke & Roberts, 1987; Renshaw, 2003; Renshaw, Chambless, & Steketee, 2006; Simon, 1988; Smith, Friedman, & Nevid, 1999; Steketee, 1987, 1993; Steketee & Chambless, 2001; Svanborg, Bäärnhielm, Åberg Wistedt, & Lützen, 2008; Telfer, 1991; Thorpe, Freedman, & Lazar, 1985; Torres, Hoff, Padovani, & Ramos-Cerqueira, 2012; Turgeon, Marchand, & Dupuis, 1998; Van Minnen & Kampman, 2000; Yen et al., 2007; Young, 1997)
No valid measure of social support (Number of excluded articles: 9)
(Addis et al., 2004; Bond & Guastello, 2013; Franklin, 1989; Hafner, 1983, 1988; Shandley et al., 2008; Tynes, Salins, Skiba, & Winstead, 1992; Xia & Hai-Yin, 2004; Yan & Cui, 2003)
No valid measure of PD/A or OCD severity (Number of excluded articles: 29)
(Albert, Maina, Saracco, & Bogetto, 2006; Chambless et al., 2001; Chambless, Floyd, Rodebaugh, & Steketee, 2007; Green, Grace, Lindy, Gleser, & Leonard, 1990; Katerndahl & Realini, 1997a; Korostil & Feinstein, 2007; Lincoln et al., 2010; Ma, Zhao, & Luo, 2007; Marchesi et al., 2014; Markowitz et al., 1989; Maulik, Eaton, & Bradshaw, 2010; McLeod, 1994; Murphy, Michelson, Marchione, Marchione, & Testa, 1998; Panayiotou & Karekla, 2013; Pankiewicz, Majkowicz, & Krzykowski, 2012; Préville et al., 2010; Priest, 2013; Renshaw, Chambless, & Steketee, 2001; Renshaw, Chambless, Rodebaugh, & Steketee, 2000; Renshaw, Chambless, & Steketee, 2003; Simmons, Gordon, & Chambless, 2005; Staebler, Pollard, & Merkel, 1993; Steketee, Lam, Chambless, Rodebaugh, & McCullouch, 2007; Takeuchi et al., 1997; Vázquez, Torres, Otero, & Díaz, 2011; Wang & Zhao, 2012; Whisman, 2007; Wood, Salguero, Cano-Vindel, & Galea, 2013; Xu, Zhao, Li, & Lü, 2000; Zaider, Heimberg, & Iida, 2010)
Not an adult sample (Number of excluded articles: 2)
(Amir, Freshman, & Foa, 2000; Grunes, Neziroglu, & McKay, 2001)
No clinical PD/A or OCD (for the whole sample or part of the sample) (Number of excluded articles: 10)
(Amazonas, Arcoverde, Caldas, & da Silva, 2010; Bland & Hallam, 1981; Brown, Harris, & Eales, 1993; Gomes et al., 2010; Kenardy, Heron-Delaney, Bellamy, Sterling, & Connelly, 2014; Kong, 2008; Landman-Peeters et al., 2005; Pinto, Van Noppen, & Calvocoressi, 2013; Powers, 1984; Sochos, 2014)
Comprehensive review (Number of excluded articles: 19)
(Bressi & Guggeri, 1996; Cobb, 1982; Côté & Gauthier, 1988; Craske & Zoellner, 1995; Fokias & Tyler, 1995; Friedman & Paradis, 2002; Goldfarb, Trudel, Boyer, & Preville, 2007; Gore & Carter, 2001; Hand, 2000; Jackson & Wenzel, 2002; Jacobson, Holtzworth-Munroe, & Schmaling, 1989; Kleiner & Marshall, 1985; Marcaurelle, Bélanger, & Marchand, 2003; Marchand, Comeau, & Trudel, 1994; Mester, 1981; Rohrbaugh & Shean, 1988; Shean, 1990; Vandereycken, 1983; Wilson, 1984)
Case report (Number of excluded articles: 2)
(Hafner, 1982; Holmes, 1982)
Not a study (treatment guide) (Number of excluded articles: 1)
(Van Noppen & Steketee, 2003)

### Support and OCD Severity

#### The Association Between OCD Severity and Marital Adjustment

Two studies looked at the association between marital adjustment and OCD. In 2006, Abbey conducted a study in order to examine romantic relationship functioning in individuals with OCD. The results indicated that relationship satisfaction negatively correlated with the obsessing (*r* = -.26, *p* < .05) and positively correlated with the neutralizing (*r* = .26, *p* < .05) subscales of the Obsessive-Compulsive Inventory, Revised (OCI-R). Self-disclosure, which is an index of positive social support, negatively correlated with the obsessive subscale of the OCI-R (*r* = -.30, *p* < .05). Moreover, the obsessive subscale also negatively correlated with the emotional subscale (“my partner listens to me when I need someone to talk to”) of the Personal Assessment of Intimacy in Relationship (PAIR; Schaefer & Olson, 1981; *r* = -.27, *p* < .05). No other significant associations between measures of marital adjustment and OCD severity were found. Riggs, Hiss, and Foa (1992) also looked at the link between marital distress and OCD symptom severity. The results indicated a significant negative correlation between marital adjustment and avoidance of the situation related to the main obsession (*r* (50) = -.28, *p* < .05), as rated by an independent assessor, but not between marital adjustment scores and ratings of main obsessions (*r* (52) = -.04, *p* > .70) or rituals (*r* (52) = .06, *p* > .65). For a summary of these results, see Table 2.

**Table 2:** Description of Studies Examining the Association Between Marital Adjustment and OCD

	Author(s)	Year	Participants	Patients’ gender	Diagnostic measure	Measure(s) of severity	Measure(s) of marital adjustment	Results
1	Abbey	2006	64 OCD patients	25 men, 39 women	OCI-R (cut-off score of 4 on the Obsessing scale)	OCI-R	PAIR, SDI, and RAS	Relationship satisfaction (RAS) correlated with the obsessing (-.26, p <.05) and neutralizing (.26, p < .05) subscales of the OCI-R. Self-disclosure correlated with the obsessing subscale of the OCI-R (-.30, p < .05). All subscales of the PAIR correlated significantly with the obsessive subscale of the OCI-R.
2	Riggs, Hiss, & Foa	1992	54 OCD patients	20 men, 34 women	NS	Assessor rating	LWMAT	Significant correlation between LWMAT and the assessor rating of avoidance (*r* (50) = -.28, p < .05), but not between LWMAT scores and ratings of main obsession (*r* (52) = -.04, p > .70) or ritual (*r* (52) = .06, p > .65).

*Note.* OCI-R = Obsessive-Compulsive Inventory-Revised; PAIR = Personal Assessment of Intimacy in Relationships; RAS = Relationship Assessment Scale; SDI = Self Disclosure Index.

#### The Association Between OCD Severity and Accommodation

Seventeen studies examined the association between accommodation and OCD. All but one study (Drury, Ajmi, de la Cruz, Nordsletten, & Mataix-Cols, 2014) found a significant association between the level of accommodation by the relatives and the severity of OCD. All of them (except for Drury et al., 2014) used the Family Accommodation Scale (FAS) and the Yale-Brown obsessive-compulsive scale (Y-BOCS), which renders them easily comparable. Given that some studies used the same sample (Albert et al., 2010; Albert, Brunatto, Maina, & Bogetto, 2009; Van Noppen & Steketee, 2009; Van Noppen, 2003), results are combined in the presentation of the data.

Many studies found a significant positive correlation between accommodation and total scores on the measure of obsessive-compulsive disorder severity (Boeding et al., 2013; Calvocoressi et al., 1999; Cherian, Pandian, Bada Math, Kandavel, & Reddy, 2013; Cherian, Pandian, Bada Math, Kandavel, & Janardhan Reddy, 2014; Ferrão & Florão, 2010; Gomes et al., 2014; Ramos-Cerqueira, Torres, Torresan, Negreiros, & Vitorino, 2008; Stewart et al., 2008; Vikas et al., 2011; Wu, Pinto, et al., 2016), meaning that the more accommodation provided by the relatives, the more severe the OCD symptoms.

Some authors also found significant association between specific areas of accommodation and symptoms of OCD. Indeed, Vikas and colleagues (2011) found that participation in rituals was correlated with the level of obsessions (*r* = .52, *p* < .01) and compulsions (*r* = .54, *p* < .01). Some results demonstrated that the level of accommodation was associated with contamination/cleaning compulsions (*r* = .18, *p* = .03, Albert et al., 2010; *r* = .26, *p* = .007, Stewart et al., 2008). Also, Albert and colleagues (Albert, Brunatto, Maina, & Bogetto, (2009; Albert et al., 2010) found that accommodation total scores were significantly correlated with obsessions (*r* = .21, *p* = .013) but not compulsions (*r* and *p* are not available in the original article). However, results from Beoding’s study (2013) indicated a significant positive correlation between accommodation and severity of compulsions (*r* = .39, *p* < .05) but not with severity of obsessions (*r* = .26, *p* > .05), which contradicts results obtained by Albert and colleagues (2010).

Other authors also found that accommodation by the family could predict the severity of OCD symptomatology. Indeed, Van Noppen and Steketee (2003, 2009) reported that the level of accommodation, as rated by patients, predicted OCD severity in regression analyses (*b* = 0.47, *p* < .01, partial correlation = .42, R^2^change = .16, *p* > .01). Accommodation was also predictive of OCD severity when rated by relatives (*b* = 0.50, *p* < .01, partial correlation = .46, R^2^change = .20, *p* > .01). Accommodation alone as rated by patients and relatives explained 16 and 20%, respectively, of the symptom severity (*F* (1,44) = 9.19, *p <* .01; *F* (1.44) = 11.7, *p* = .001). They also found that among many factors (e.g. relatives’ attributions, emotional over-involvement, and criticism), accommodation was the strongest predictor of OCD severity, explaining 42% of the variance (direct causal effects .42, *p* < .05). On the other hand, other authors found that OCD symptomatology could predict accommodation behaviors among the relatives. For example, Stewart and colleagues (2008) reported significant correlations between OCD severity and cleaning/contamination symptoms (*r* = .26, *p* = .007). When entered in a stepwise regression analysis, these factors remained significant. Similarly, Albert and colleagues (2010) also entered the significant factors in a regression analysis and reported that a higher FAS total score was predicted by the contamination/cleaning symptom dimension score (*β* = 0.22, *t* = 2.87, *p* = .005).

The only study that found negative results is the one by Drury and colleagues (2014). They conducted a study on hoarders and their relatives in order to assess the impact of hoarding on functioning as well as burden for the relatives. They used a different measure, the Family Impact Scale for Hoarding disorder (FISH), in order to assess both the level of family accommodation displayed by the relatives and the associated burden. The results indicate that hoarding severity did not predict FISH scores (*b* = 0.20, *t* = 1.33, *p* = .190).

Finally, two recent meta-analyses investigated the association between family accommodation and OCD severity. In Strauss, Hale, and Stobie (2015), results from 14 studies (seven with adults and seven on pediatric OCD) showed a statistically significant medium effect size (*r* = .35, 95% CI [.23, .47]), so that family accommodation accounts for approximately 12% of the variance in OCD symptom severity. In Wu, McGuire, et al. (2016), 41 studies on accommodation and OCD severity were included. Among those studies, 15 were on adults with OCD. Results showed a medium positive effect (*r* = .42, 95% CI [.36, .47], *z* = 13.00, *p* < .001), which indicates that higher OCD severity is associated with increased family accommodation. Also, there was no significant difference by categorical age groups (*Q*(1)__btwn__ = 1.36, *p* = .24) and no significant effect when examining participant mean age (*β* = -0.002, *SE* = .003, *z* = -0.82, *p* = .41). This suggests that the association between family accommodation and OCD severity is similar whether participants are adults or children. For a summary of these results, see Table 3.

**Table 3:** Description of Studies Examining the Association Between Accommodation and OCD

	Author(s)	Year	Participants	Patients’ gender	Diagnostic measure	Measure(s) of severity	Measure(s) of accommodation	Results
1	Albert, Brunatto, Maina, & Bogetto	2009, 2010	97 OCD patients and 141 relatives	49 men, 48 women	SCID-I	Y-BOCS	FAS	FAS and Y-BOCS total scores were positively correlated. FAS total score was significantly correlated with Y-BOCS obsession subscale.
2	Boeding	2013	20 OCD patients and their partner	1 man, 19 women	MINI	Y-BOCS	FAS	FAS and Y-BOCS total scores were positively correlated (.39, *p* < .05). FAS total score was significantly correlated with Y-BOCS compulsion subscale (.39, *p* < .05).
3	Calvocoressi et al.	1999	36 OCD patients and 36 relatives	19 men, 17 women	Evaluation by a psychiatrist	Y-BOCS	FAS	FAS and Y-BOCS total scores were positively correlated (*r* = .49, *p* < .003, n = 34).
4	Cherian, Pandian, Badamath, Kandavel, & Reddy	2013	100 OCD patients and their primary caregiver	57 men, 43 women	NS	Y-BOCS	FAS	FAS and Y-BOCS total scores were positively correlated.
5	Cherian, Pandian, Badamath, Kandavel, & Reddy	2014	94 OCD patients and their primary caregiver	52 men, 42 women	MINI	CGI and Y-BOCS	FAS	Y-BOCS total scores (*r* = .30, *p* < .01) and CGI (*r* = .34, *p* < .001) were correlated with FAS total scores.
6	Drury, Ajmi, de la Cruz, Nordsletten, & Mataix-Cols	2014	41 OCD patients (hoarding disorder) and 60 relatives with hoarding	9 men, 32 women	SIHD	HRS-SR	FISH	OCD severity did not predict FISH scores (*b* = 0.20, *t* = 1.33, *p* = .19)
7	Ferrão & Florão	2010	47 OCD patients and 47 relatives	22 men, 25 women	SCID-I	Y-BOCS	FAS	FAS and Y-BOCS total score (.71, *p* < .001, n = 45)
8	Gomes et al.	2014	114 OCD patients and 114 relatives	43 men, 71 women	SCID-I	CGI, OCI-R, and Y-BOCS	FAS-IR	Positive correlations (spearman) between FA and Y-BOCS obsessions (*r* = .28, *p* = .002), compulsions (*r* = .26, *p* = .005) and total scores (*r* = .30, *p* = .001). Positive correlation between FA and CGI (*r* = .34, *p* < .001).
9	Ramos-Cerqueira, Torres, Torresan, Negreiros, & Vitorino	2008	50 OCD patients and 50 caregivers	22 men, 28 women	NS	Y-BOCS	FAS	Positive correlation between Y-BOCS and FAS total scores (*r* = .26, *p* < .001).
10	Stewart et al.	2008	110 OCD patients and 110 relatives	58 men, 52 women	Assessment by both a psychiatrist and a behavior therapist	Y-BOCS	FAS	Y-BOCS and FAS total scores were positively correlated (*r* = .35, *p* = .0003). FAS was associated with cleaning/contamination compulsions (*r* = .26, *p* = .007)
11	Strauss, Hale, & Stobie (meta-analysis)	2015	14 included studies (7 on adult OCD) 849 OCD patients and 849 relatives	38% to 57% female	DSM-IV criteria, Y-BOCS (score 16+), DCR-10	Y-BOCS	FAS	The medium effect size was significant (r = .35; 95% CI: .23 to .47). Family accommodation accounts for approximately 12% of the variance in OCD symptom severity.
12	Van Noppen & Steketee	2003, 2009	50 OCD patients and 50 relatives	23 men, 27 women	SCID-I	Y-BOCS	FAS	FA made the largest contribution in the model, explaining 42% of the variance in OCD severity (direct causal effects .42, *p* < .05).
13	Viskas, Avasthi, & Sharan	2011	32 OCD patients and 32 relatives	NS (Majority of patients were male)	DCR-10	Y-BOCS	FAS	Participation in rituals was positively correlated with Y-BOCS obsession (*r* = .52, *p* < .01), compulsion (*r* = .54, *p* < .01) and total score (.55, *p* < .01). Total FA was positively correlated with all subscales of the Y-BOCS (obsession: *r* = .49, *p* < .01; compulsion: *r* = .50, *p* < .01; total: *r* = .51, *p* < .01).
14	Wu et al.	2016	61 OCD patients and 54 relatives (18 were spouses)	27 men, 34 women	Clinical consensus between researcher and psychologist	Y-BOCS, CGI	FAS-PV	FA, as perceived by patients, correlated positively with Y-BOCS (*r* = .37, *p* < .01) and CGI (*r* = .53, *p* < .001).
15	Wu et al. (meta-analysis)	2016	41 included studies (15 on adult OCD) 2509 OCD patients	50% female	NS	NS	NS	The random effects meta-analysis identified a medium positive effect, (r = .42, 95% CI [.36, .47], z = 13.00, p < .001).

*Note.* CGI = Clinical Global Impression; DCR = ICD-10 Diagnostic Criteria for Research; FA = Family Accommodation; FAS = Family Accommodation Scale; FAS-IR = Family Accommodation Scale-Interviewer Rated; FAS-PV = Family Accommodation Scale-Patient Version; FISH = Family Impact Scale for Hoarding Disorder; HRS-SR = Hoarding Rating Scale-Self Report; MINI = Mini-International Neuropsychiatric Interview; OCI-R = Obsessive Compulsive Inventory- Revised ; SCID-I = Structured Clinical Interview for DSM-IV Axis I disorders; SIHD = Structured Interview for Hoarding Disorders; Y-BOCS = Yale-Brown Obsessive Compulsive Scale.

#### The Association Between OCD Severity and Expressed Emotion

Three studies were interested in the association between OCD severity and Expressed Emotion, with one of them demonstrating a significant association. Indeed, Cherian and colleagues’ (2014) results indicate that perceived criticism was associated with OCD severity as measured by the Y-BOCS (*r* = .24, *p* < .01) and the Clinical Global Impression (CGI; *r* = .27, *p* < .01). Finally, Van Noppen and Steketee (2003, 2009) found no significant association between Expressed Emotions variables (criticism, hostility, emotional over-involvement) and OCD severity, when rated by patients or relatives. When accommodation of the family was entered in the model, criticism lost its significance (Van Noppen & Steketee, 2003, 2009). See Table 4 for a summary of the results.

**Table 4:** Description of Studies Examining the Association Between Expressed Emotion and OCD

	Author(s)	Year	Participants	Patients’ gender	Diagnostic measure	Measure(s) of severity	Measure(s) of Expressed Emotion	Results
1	Cherian, Pandian, Badamath, Kandavel, & Reddy	2014	94 OCD patients and their primary caregiver	52 men, 42 women	MINI	CGI and Y-BOCS	FEICS (patient rated)	Perceived criticism (FEICS) was associated with Y-BOCS total score (*r* = .24, *p* < .01) and CGI (*r* = .27, *p* < .01).
2	Van Noppen & Steketee	2003 2009	50 OCD patients and 50 relatives	23 men, 27 women	SCID-I	Y-BOCS	IRQ, LEE, PCM, PRS, and RRQ	No significant association between EE variables (criticism, hostility) and OCD severity, when EE was rated by patients or relatives.

*Note.* BAT = Behavioural Avoidance Test; CFI = Camberwell Family Interview; CGI = Clinical Global Impression; FEICS = Family Emotional Involvement and Criticism Scale; IRQ = Influential Relationships Questionnaire; LEE = Level of Expressed Emotion Scale; MINI = Mini-International Neuropsychiatric Interview; PCM = Perceived Criticism Measure; PRS = Patient Rejection Scale; RRQ = Relative’s Reaction Questionnaire; SCID-I = Structured Clinical Interview for DSM-IV Axis I disorders; SCID-P = Structured Clinical Interview for DSM-III-R – Patient version; TSR = Target Symptom Ratings

### Support and PD/A Severity

#### The Association Between PD/A Severity and Social Support

Three studies looked at the link between PD/A and social support, with two of them demonstrating that the level of support is associated with the severity of PD/A symptoms. Huang and colleagues (2010) developed a structural equation model in order to investigate the effects of social support on panic and agoraphobic symptoms as well as suicidal ideation. They found that social support influenced panic symptoms (negative association, -.47), which then influenced agoraphobic symptoms (*χ*^2^_8_ = 3.53; AGFI = 0.95; *p* = .897). Smith (1998) found a significant negative correlation between the size of support (number of friends) and the frequency of panic attacks (*r* (66) = -.25, *p* = .04) in a sample of African Americans. Although social support appraisal did not predict agoraphobia in a regression analysis, the authors did find a significant negative correlation between the two variables (*r =* -.27, *p* < .05). However, they did not find a link between social support appraisal and severity of panic symptoms.

Renneberg, Chambless, Fydrich, and Goldstein (2002) conducted a study in order to investigate affect balance in dyads of patients and their relatives and its association with outcome following cognitive behavioral therapy. Given that the relatives were nine parents and 26 spouses, this study was included under both social and marital support (see paragraph below). In order to assess the level of affectivity in their sample, the authors used an observational measure, which assesses both verbal and non-verbal behaviors in an interaction between partners. Based on these observations, they separated the group between affect-balanced and affect-unbalanced dyads. The authors found that the two groups did not differ on pre-treatment scores of measures of agoraphobia and panic (*t*-tests *p* > .17 for all measures), which means that the quality of the interaction during a problem-solving task between partners was not associated with symptoms severity. Refer to Table 5 for a summary of these results.

**Table 5:** Description of Studies Examining the Association Between Social Support and PD/A

	Author(s)	Year	Participants	Patients’ gender	Diagnostic measure	Measure(s) of severity	Measure(s) of social support	Results
1	Huang, Yen, & Lung	2010	60 PDA patients	30 men, 30 women	MINI	PASC	SSS	Social support was a direct protector of panic symptoms (-.47), but not agoraphobic symptoms
2	Renneberg, Chambless, Fydrich, & Goldstein	2002	35 PDA patients and 35 significant others (26 spouses, 9 parents)	12 men, 23 women	SCID-I	MIA	KPI	Groups of affect-balanced versus affect-unbalanced dyads did not differ at pretreatment on measures of agoraphobia and panic (*t*-tests, all *p*s > .17)
3	Smith	1998	81 PDA patients	7 men, 74 women	ADIS-IV	BSQ, FQ, and MIA	SSAS and SSRS	Significant correlation between size of support – friends and frequency of panic/month (*r* (66) = -.25, *p* = .04).

*Note.* ADIS-IV = Anxiety Disorder Interview for DSM-IV; BSQ = Body Sensations Questionnaire; FQ = Fear Questionnaire; KPI = Kategoriensystem für Parnerschaftliche Interaktion (interaction coding system); MIA = Mobility Inventory for Agoraphobia; MINI = Mini-International Neuropsychiatric Interview; PASC = Panic and Agoraphobic Symptoms Checklist; SCID-I = Structured Clinical Interview for DSM-III-R; SSS = Social Support Scale; SSAS = Social Support Appraisals Scale; SSRS = Social Support Resources Scale.

#### The Association Between PD/A Severity and Marital Adjustment

Eleven studies evaluated the link between marital adjustment and the severity of either panic disorder and\or agoraphobia. Authors reported mixed results. Since authors did not all use the same measure of marital adjustment, results will be presented by the type of measure utilized in order to compare similar articles. It is thus possible that one study gets described in several places due to its use of multiple questionnaires.

##### Self-report measures

Three studies used the Maudsley Marital Questionnaire (MMQ) or its modified version (MMMQ) as a measure of marital adjustment. More specifically, the MMQ assesses three domains: marital adjustment, sexual adjustment and general life with the partner (e.g. domestic task, social activity). None of the studies found a significant correlation between marital adjustment and panic or agoraphobia symptoms. Indeed, Arrindell, Emmelkamp, and Sanderman (1986) found no significant correlation between the MMQ marital scale and severity, as assessed by the Fear Questionnaire (FQ) and an observation of phobic anxiety and avoidance by both the therapist and an independent observer (Watson & Marks, 1971; *p* > .02 for all measures (Bonferroni adjustment)). Cobb, Mathews, Childs Clarke, and Blowers (1984) evaluated whether integrating the spouse as a co-therapist would enhance the outcome of a behavioural therapy for agoraphobia. The authors found that there was no association between initial severity of marital problems, as assessed by the MMMQ, and the severity of agoraphobia. However, no statistics were presented for this result. Monteiro, Marks, and Ramm (1985) also used the MMMQ and found that at pre-treatment, there was no significant difference in agoraphobic symptoms, as assessed by the FQ, between participants qualified as being in a “good” versus a “less good” marriage (*p* > .05). Although not explicitly reported by the authors of the original study, the authors of the present review based their results on graphs presented in the original study.

Chambless (1985) used the Marital Dissatisfaction Questionnaire (MDQ), which is a five-item questionnaire that assesses the discrepancy between the respondent’s perception of his/her actual and ideal spouse. The author did not find any significant correlations between the level of marital dissatisfaction and severity of agoraphobia (*r* = .10, *p* > .05) or frequency of panic attacks (*r* = -.10, *p* > .05).

Marcaurelle, Bélanger, Marchand, Katerelos, and Mainguy (2005) were interested to see the effects of marital conflicts and adjustment on severity of PDA. They used the Dyadic Adjustment Scale (DAS), which is a measure that assesses four areas of marital adjustment: cohesion, consensus, satisfaction and affection. Their results demonstrate that patients with PDA who demonstrated lower levels of marital adjustment had more frequent catastrophic thoughts (*r* = -.048, *p* < .0001) as well as stronger fear of bodily sensations (*r* = -.33; *p* < .007) and fear of consequences of anxiety (*r* = -.46, *p* < .002). No significant correlation was found between PDA total clinical severity and marital adjustment. El-Baalbaki and colleagues (2011) were also interested in marital interactions as a predictor of panic and agoraphobia symptom severity. Comparable to the results of Marcaurelle and colleagues (2005) described above, they found significant negative correlations between DAS and catastrophic thoughts (*r* = -.46, *p* < .01), fear of bodily sensations (*r* = -.31, *p* < .05), and fear of consequences of anxiety (*r* = -.49, *p* < .01). However, Peter, Hand, and Wilke (1993) found no association between agoraphobic severity, as measured by the FQ, and DAS scores.

In their study, Lange and Van Dyck (1992) utilized the Interactional Problem Solving Inventory (IPSI), which is a self-report questionnaire that measures the extent to which partners are satisfied with their problem-solving abilities. They did not find any significant correlation between relationship quality and agoraphobic severity before treatment, except for the avoidance of busy streets subscale of the FAS-IR (*r* = -.29, *p* < .10). However, the significance level was set at .10 and there is no mention of whether or not the test was one-tailed or two-tailed.

Finally, Tukel (1995) divided his 45 participants with PDA into three subgroups, those of housewives, working women, and working men. Participants were assessed on severity of PDA (FQ) and quality of marital relationship (MMQ). Results indicated a significant positive correlation between severity of PDA and quality of marital relationship for housewives *(r* = .61, *p* = .04). No significant correlations were found for the other subgroups (*r* = .15, *p* > .05 for working women; *r* = .10, p > .05 for working men).

##### Observational measures

Two studies included an observational measure of the interaction between patients and their relatives. Chambless and colleagues (2002) were interested in the marital interaction between couples in which one partner has PDA and a control group. They used the Kategoriensystem für Parnerschaftliche Interaktion (KPI), which is a system used to code a problem-solving interaction between two partners. During analysis of the interaction, each meaningful unit of speech is assigned a verbal and non-verbal code (e.g. positive, negative, or neutral). The authors found that panic frequency was not significantly related to any self-reported marital variables. However, they demonstrated that husbands whose wives were more avoidant engaged in a higher rate of negative verbal behavior (*r* = .44, *p* < .006) and were more critical (*r* = .35, *p* < .031) during the problem-solving interaction. The Renneberg and colleagues’ study (2002), described in a section above, also used the KPI. They did not find a significant difference between marital adjustment and severity of panic or agoraphobic symptoms.

El-Baalbaki and colleagues (2011) used a different observational measure, the Global Couple Interaction Coding System (GCIS). It also evaluates partners during a problem-solving situation but it evaluates each partner on five components of their verbal and non-verbal marital interaction. The five components are divided into three negative dimensions: (a) avoidance of and withdrawal from the discussion, (b) dominance, asymmetry in the control of the conversation, and (c) hostility, criticism, and conflict; and two positive dimensions: (a) support and validation, which reflect active listening and warmth, and (b) problem-solving skills. Behaviors are rated according to four levels of severity (absent, mild, moderate, excessive). The authors found many significant correlations between aspects of the interaction and symptoms of panic and agoraphobia. Indeed, spouse’s criticism and hostility were positively correlated with fears of bodily sensations (*r* = .31, *p* < .05) and catastrophic thoughts (*r* = .39, *p* < .01). Spouse’s dominance was also positively correlated with these two variables (*r* = .29, *p* < .05 and *r* = .38, *p* < .01, respectively). Spouse’s support-valid action was negatively correlated with PDA clinical severity (*r* = -.26, *p* < .05), catastrophic thoughts (*r* = -.31, *p* < .05), and agoraphobic avoidance (when accompanied; *r* = -.31, *p* < .05). Spouse’s problem-solving skills and clarification/negotiation were negatively associated with agoraphobic avoidance (when accompanied; *r* = -.26, *p* < .05). Lastly, quality of solutions by the spouse was negatively associated with fear of bodily sensations (*r* = -.25, *p* < .05). For further information, refer to Table 6.

**Table 6:** Description of Studies Examining the Association Between Marital Adjustment and PD/A

	Author(s)	Year	Participants	Patients’ gender	Diagnostic measure	Measure(s) of severity	Measure(s) of marital adjustment	Results
1	Arrindell, Emmelkamp& Sanderman	1986	23 PDA patients and their partner	23 women	Clinical interview	FQ, *Phobic Anxiety* and *Phobic Avoidance* ratings by therapist and independent observer	Clinical interview by an independent assessor, and MMQ	No significant correlation between any measure of PDA severity and measures of marital quality (all *p*s > .02).
2	Chambless	1985	378 PDA patients	64 men, 314 women	Diagnostic interview	MI	MDQ	No statistical correlation between MDQ and severity of agoraphobia (*r* = .10, *p* > .05, n = 74) or frequency of panic attacks (*t* = -.10, *p* > .05, n =108)
3	Chambless et al.	2002	22 PDA patients and their partner	22 women	SCID-I	MIA	KPI	Husbands whose wives were more avoidant engaged in a higher rate of negative verbal behavior (*r* = .44, *p* = .006) and were more critical (*r* = .35, *p* = .031). Panic frequency was not significantly related to any marital variable.
4	Cobb, Mathews, Childs-Clarke, & Blowers	1984	19 agoraphobic patients and their partner	4 men, 15 women	Diagnostic interview	FQ	MMMQ	No assciation between the initial severity of marital problems and the severity of agoraphobia.
5	El-Baalbaki et al.	2011	65 PDA patients and their partner	19 men, 46 women	ADIS-IV-L	ACQ, ADIS-CSR, ASI, BSQ, MIA	DAS and GCIS	PDA severity correlated negatively with positive behaviors and positively with negative behaviors during the problem-solving interaction. Marital adjustment, as reported by PDA patients, was correlated with BSQ (*r* = -.31, *p* < .05), ACQ (*r* = -.46, *p* < .01), and ASI (*r* = -.49, *p* < .01) scores.
6	Lange & Van Dyck	1992	25 PDA patients	NS	NS	FAS-IR and FQ	IPSI	No significant correlation between agoraphobic severity and problem solving.
7	Marcaurelle, Bélanger, Marchand, Katerelos, & Mainguy	2005	67 PDA patients	23 men, 44 women	ADIS-IV-L	ADIS-CSR, ASI, BSQ, MIA	DAS	Marital adjustment was associated with ACQ (*r* = -.48, *p* < .0001), BSQ (*r* = -.33; *p* < .007) and ASI (*r* = -.46, *p* < .002) scores.
8	Monteiro, Marks, Ramm	1985	27 agoraphobic patients	4 men, 23 women	NS	FQ	MMMQ	Subjects with good and less good marriages did not differ at pretreatment on agoraphobic severity.
9	Peter, Hand, & Wilke	1993	25 agoraphobic patients and their partner	3 men, 22 women	Evaluation by a psychiatrist, according to DSM-III criteria	FQ	DAS	No association between severity of agoraphobia and marital adjustment.
10	Renneberg Chambless, Fydrich, & Goldstein	2002	35 PDA patients and 35 significant others (26 spouses, 9 parents)	12 men, 23 women	SCID-I	MIA and panic frequency/week	KPI	Groups of affect-balanced versus affect-unbalanced dyads did not differ at pretreatment on measures of agoraphobia and panic (*t*-tests, all *p*s > .17).
11	Tukel	1995	45 PDA patients	15 men, 30 women	DSM-III criteria	FQ	MMQ	Quality of relationship was positively correlated with PDA severity for housewives (*r* = .61, *p* = .04). No other significant correlation.

*Note.* ACQ = Agoraphobic Cognitions Questionnaire; ADIS-IV-L = Anxiety Disorders Interview Schedule, Lifetime Version; ADIS-CSR = Anxiety Disorders Interview Schedule- Clinician Severity Rating; ASI = Anxiety Sensitivity Inventory; BSQ = Body Sensations Questionnaire; DAS = Dyadic Adjustment Scale; FAS = Fear and Avoidance Scales- Interviewer Rated; FMSS = Five-Minute Speech Sample; FSS = Fear Survey Schedule; FQ = Fear Questionnaire; GCIS = Global Couple Interaction Coding System; IPSI = Interactional Problem Solving Inventory; KPI = Kategoriensystem für Partnerschaftliche Interaktion (interaction coding system); LWMAT = Lock and Wallace Marital Adjustment Test; MI = Mobility Inventory; MIA = Mobility Inventory for Agoraphobia; MDQ = Marital Dissatisfaction Questionnaire; MMQ = Maudsley Marital Questionnaire; MMMQ = Modified Maudsley Marital Questionnaire; MQ = Marital Questionnaire; NS = Not specified; PCS = Perceived Criticism Scale; SCID-I = Structured Clinical Interview for DSM-III-R disorders.

#### The Association Between PD/A Severity and Expressed Emotion

Two studies assessed the level of Expressed Emotion in relation to the severity of agoraphobia. Peter and colleagues (1993) reported significant associations between the severity of agoraphobia and the critics and emotional warmth subscales of the CFI (critics: *r* = .55, *p* < .01; emotional warmth: *r* = -.56, *p* < .01). In Rodde and Florin's (2002) study, 46 participants with PD/A and their partner were included. However, only results for 32 couples were reported (14 couples dropped out). Expressed Emotion status was assessed with the Five-Minute Speech Sample (FMSS), a five-minute monologue during which the partner is asked to talk about the patient and their relationship. There were no significant associations between Expressed Emotion status and fear of bodily sensations (BSQ). For a summary, see Table 7.

**Table 7:** Description of Studies Examining the Association Between Expressed Emotion and PD/A

	Author(s)	Year	Participants	Patients’ gender	Diagnostic measure	Measure(s) of severity	Measure(s) of Expressed Emotion	Results
1	Peter, Hand, & Wilke	1993	25 agoraphobic patients and their partner	3 men, 22 women	Evaluation by a psychiatrist, according to DSM-III criteria	FQ	CFI	Severity was positively associated with criticism (*r* = .55, p < .01) and negatively with emotional warmth (*r* = -.56, p < .01) as perceived by the patient.
2	Rodde & Florin	2002	32 PD/A patients and their partner	12 men, 20 women	DSM-III criteria	BSQ	FMSS	No significant association (statistics not provided by authors from original study).

*Note.* BSQ = Body Sensations Questionnaire; CFI = Camberwell Family Interview; FQ = Fear Questionnaire

## Discussion

### OCD Studies

Suffering from OCD can create major changes in the dynamics of an intimate relationship and the family. Results presented above generally demonstrate that the level of support influence the severity of OCD. Indeed, all but one study assessing family accommodation found significant results, indicating that the more accommodation behaviors performed by the relatives, the more severe the OCD symptoms. Both meta-analyses also found a positive association between family accommodation and OCD severity. However, half of the sample of studies in the Strauss et al. (2015) meta-analysis were studies on pediatric OCD. Given that the authors did not examine the effect of age, it cannot be concluded that results would have been the same for adult patients only.

Although family members wish to alleviate the burden on the patients by modifying their habits and participating in the rituals, their behaviors seem to maintain and contribute to the severity of the disorder by favouring avoidance by the patients. It is also interesting to note that the authors of this study consider accommodation as a specific measure of social support because it is considered as support that is directly linked to the symptoms of OCD. A systematic review by Fredette and colleagues (2016) also found that PTSD specific measures of social support tended to be more associated with the outcome of cognitive behavioral therapy than global measures of support. Results relating to Expressed Emotion and OCD severity are mixed. Cherian and colleagues (2014) found that the level of criticism influenced OCD severity so that victims with relatives who are more critical of them seem to experience more severe symptoms. Van Noppen and Steketee (2009) tested a model combining accommodation and measures of Expressed Emotion. Criticism was indeed correlated to the severity of OCD. However, measures of Expressed Emotion did not predict severity when accommodation was considered. This latter result supports the other studies, which found a robust link between accommodation and OCD severity. Finally, marital adjustment also seems to be associated with OCD severity, so that a better quality of relationship between partners is correlated with less severe symptoms. These results are based on two studies.

### PD/A Studies

Studies analyzing the association between social or marital support and the severity of PD/A present mixed results. Researches using measures of social support seem to indicate that people with good support, either in terms of their perception of the quality of their support or the size of their network, present less severe symptoms. Concerning marital adjustment, results are mixed and the methods used were diverse. Nine studies used self-reported measures, from which two found that better marital adjustment was negatively correlated with symptoms of panic and agoraphobia. Interestingly, these two studies used the same questionnaire, the DAS, and found strikingly similar results. Another study found a difference between satisfied and unsatisfied dyads on measures of symptom severity. However, significance was not assessed. Surprisingly, one study (Tukel, 1995) also found that marital adjustment between housewives with PDA and their spouses was positively correlated with severity of disorder. There were no significant correlations between marital adjustment and PDA severity among working men and women. These results may be understood using the assortative mating hypothesis, which suggests that partners choose each other on the basis of perceived attributes (Hafner, 1977). For example, a woman with agoraphobia who has dependent traits may choose a partner with more dominant traits. Both partners would thus benefit from a dynamic where the husband endorses more responsibilities and takes care of his agoraphobic wife. Given that improvement in agoraphobic symptoms would lead to more autonomy from the wife and break this dynamic, it may also lead to a decline in marital satisfaction for both partners. Thus, it is possible that housewives and their husbands are more likely to have these attributes that allow them to benefit from an agoraphobic dynamic, in comparison to working men and women.

However, when patients are not distinguished according to their working status, most results seem to indicate that level of marital adjustment, as assessed by self-reported questionnaires, is not associated with the severity of panic and agoraphobic symptoms. Given that marital adjustment is a concept that is larger than marital support, it would be interesting to create more specific measures of marital support in order to eliminate factors that are not directly in link with support (e.g. sexuality).

Three studies also assessed marital adjustment with observational measures, two of which found significant results. These results indicated that negative social support (e.g. criticism and dominance) is associated with more severe symptoms while positive support (e.g. proposing positive solutions) is associated with less severe symptoms. However, one other study (Renneberg et al., 2002) found no significant differences between balanced-affected and unbalanced-affected dyads on measures of panic and agoraphobia severity. In order to understand the latter result, we performed statistical analyses to determine the effect sizes and statistical power, using descriptive data from Table 2 in the original article. Effect sizes were calculated between the balanced- and unbalanced-affected dyads on measures of agoraphobia and panic frequency. A medium effect size (Cohen’s *d* = .48) was found for panic frequency. However, statistical power was low (27%), hence one cannot conclude that results between the two groups on measures of panic frequency are equivalent. More studies would thus be needed to have a clearer picture.

Finally, two studies analyzed the link between Expressed Emotion and severity of PD/A. In the first study (Peter et al., 1993), it was found that criticism was negatively associated with the symptomatology of PD/A. Also, positive aspects of support were assessed with the CFI, and it was found that emotional warmth was associated with less severe symptoms. Given that Expressed Emotion assesses relatives’ attitudes and behaviors toward the patient’s illness, it is considered a specific measure of social support. As reported for the results concerning OCD, the specific way people deal with their partner’s symptoms seems to be correlated with the severity of the symptomatology. In the second study (Rodde & Florin, 2002), no significant association was found between Expressed Emotion and the severity of PD/A. However, no statistics were presented, which makes it difficult to draw conclusions.

### Conclusion

Results presented in this systematic review generally indicate that social and marital support is associated with the severity of OCD and PD/A, which supports our hypotheses. Indeed, negative social support seems to be associated with more severe symptoms whereas positive social support might be beneficial for people suffering from OCD or PD/A. These results stress the importance of relatives in helping people recover from their illness. Living with someone suffering from a mental illness can be difficult for the relatives, as they might not know how to support or react to the patient’s behaviors. Thus, solutions such as integrating the relatives in the therapy as well as providing them with tools (e.g. psychoeducation, personalized therapy for the relatives) on how to deal with the symptoms of the disorder might be beneficial to both the patient and the relatives. However, more studies would be needed to assess the level of support, using both observational and self-report measures of social support, as well as more specific measures as they seem to be more strongly correlated with the severity of OCD and PD/A. Moreover, studies that assess and compare both the negative and positive forms of social support would be interesting, since negative social support has been found to be more strongly correlated with symptom severity in a study of post-traumatic stress disorder (Zoellner, Foa, & Brigidi, 1999).

To conclude, this systematic review has some limitations. Indeed, the wide spectrum of questionnaires used by different researchers rendered it difficult to compare studies adequately. Moreover, the authors of the present study did not always have full access to the description of the questionnaires, which at times made it necessary to infer their content (e.g. MDQ). Moreover, the authors decided to cover a broad spectrum of concepts relating to social support (e.g. accommodation and marital adjustment) in order to render this systematic review as exhaustive as possible. Readers need to keep this in mind when interpreting the results. In order to compensate for this, the authors tried to only present results pertaining to social support when it was possible. For example, only FAS total scores and results of the participation in the rituals subscale were used, since other subscales concerning the impact of accommodation on relatives were not manifestations of social support as it was defined in this review. Also, results concerning emotional over-involvement were left out, since it is not included in our definition of social support. Finally, some studies had limited statistics, which makes the interpretation of their results difficult.
